# Stimulation of the basal and central amygdala in the mustached bat triggers echolocation and agonistic vocalizations within multimodal output

**DOI:** 10.3389/fphys.2014.00055

**Published:** 2014-03-04

**Authors:** Jie Ma, Jagmeet S. Kanwal

**Affiliations:** ^1^Department of Physiology and Biophysics, Georgetown UniversityWashington, DC, USA; ^2^Department of Neurology, Georgetown UniversityWashington, DC, USA

**Keywords:** amygdala, communication, complex sounds, microstimulation, autonomic activity, vocalization, fear, bat

## Abstract

The neural substrate for the perception of vocalizations is relatively well described, but how their timing and specificity are tightly coupled with accompanying physiological changes and context-appropriate behaviors remains unresolved. We hypothesized that temporally integrated vocal and emotive responses, especially the expression of fear, vigilance and aggression, originate within the amygdala. To test this hypothesis, we performed electrical microstimulation at 461 highly restricted loci within the basal and central amygdala in awake mustached bats. At a subset of these sites, high frequency stimulation with weak constant current pulses presented at near-threshold levels triggered vocalization of either echolocation pulses or social calls. At the vast majority of locations, microstimulation produced a constellation of changes in autonomic and somatomotor outputs. These changes included widespread co-activation of significant tachycardia and hyperventilation and/or rhythmic ear pinna movements (PMs). In a few locations, responses were constrained to vocalization and/or PMs despite increases in the intensity of stimulation. The probability of eliciting echolocation pulses vs. social calls decreased in a medial-posterior to anterolateral direction within the centrobasal amygdala. Microinjections of kainic acid (KA) at stimulation sites confirmed the contribution of cellular activity rather than fibers-of-passage in the control of multimodal outputs. The results suggest that localized clusters of neurons may simultaneously modulate the activity of multiple central pattern generators (CPGs) present within the brainstem.

## Introduction

The temporal structure and timing of vocalizations is critical for both echolocation and social communication. This allows vocal activity to effectively coordinate relevant outputs for goal directed behavior. In bat species, auditory vigilance is essential for successful insect capture (Ghose and Moss, 2006) as well as when conspecifics engage in many of the well-defined social behaviors (Clement and Kanwal, 2012). For example, either aggressive or distress vocalizations are precisely timed to match fight or flight behaviors as well as postures indicative of the state of vigilance (Clement and Kanwal, 2012). Within the mammalian brain, the amygdala plays an important role in driving vigilance as well as other fearful defensive behaviors, particularly social behaviors (Newman, 1999; Goodson and Kabelik, 2009; O'Connell and Hofmann, 2011; Hall et al., 2013) and in decision making (O'Connell and Hofmann, 2012) that shape an individual's responses to its physical and social environment (Quirk et al., 1995; Isenberg et al., 1999; Amaral, 2003; Machado and Bachevalier, 2006). Fear, aggression, and vigilance induce motivational shifts and enhance somatomotor and sympathetic activity to cope with predictive ambiguity in the face of imminent danger. Thus, vigilance is frequently accompanied by changes in heart and/or ventilation rates (Applegate et al., 1983; McDonald, 1998). The rapid and coordinated vocal, behavioral, and physiological responses during aggression and fear-mediated interactions between conspecifics underscore the need for a high level control for synchronizing these otherwise independently controlled, multimodal outputs. Specifically, multimodal outputs may include changes in heart rate (HR), ventilation rate (VR) and activation of somatic activity, such as pinna movements (PMs) as well as vocal behavior.

The neural parameters and organization for invoking multiple outputs, including social vocalizations, have not been systematically examined at the level of the amygdala. In the mustached bat, *Pteronotus parnellii*, social calls associated with aggressive interactions trigger transient as well as persistent activity within neurons in the basoloateral amygdala (Naumann and Kanwal, 2007, 2011; Peterson and Wenstrup, 2012). This is consistent with the presence of anterograde labeling in this region of the amygdala after injection of tracers into physiologically identified areas of the auditory cortex in mustached bats (Fitzpatrick et al., 1998). It is generally understood that sensory information flows from the lateral amygdala (LA) to the basal and central amygdala (BA and CA, respectively), although short paths from the LA to the CA, bypassing the BA, and reciprocal connections between these and other nuclei also exist (Pitkanen et al., 1997). It is also known that the CA projects to the periaqueductal gray (PAG) and paralemniscal area (PLA) in the midbrain and has reciprocal connections with the hypothalamus (Ono et al., 1985; Rizvi et al., 1991). The CA's role in an audiovocal communicative context was first studied in a primate species and recently confirmed in the mustached bat (see Ma et al., 2009 for a preliminary report).

The relatively large amygdala in the mustached bat responds robustly to communication sounds (Naumann and Kanwal, 2011). With respect to its role in eliciting vocalizations, the null hypothesis implies that neurons within the BA and/or CA are no different than other non-vocal regions and cannot elicit behaviorally relevant multimodal outputs, including vocalizations. Alternatively, these neurons may have the capacity to trigger complex vocalizations along with other physiologically relevant changes. To test these possibilities, we conducted detailed mapping using electrical microstimulation with constant current pulses delivered at near threshold levels. Simultaneous monitoring of multiple response modalities including vocalizations during focal stimulation of either the BA or CA in intact awake animals has not been attempted and little is known about their role in triggering vocal activity. To rule out the possibility of incorrectly rejecting the null hypothesis (Type I error), we also performed pharmacological manipulation with application of kainic acid (KA) at randomly targeted sites within the BA and CA.

The results reported here demonstrate a widespread capacity of amygdala neurons to elicit complex, naturalistic vocalizations. While the context-sensitivity of these vocalizations was not tested in this study, our data provide the first demonstration that multimodal control is widespread within the amygdala, suggesting that vocal behavior triggered by neural activity here has physiological and possibly behavioral consequences, and therefore would benefit from being context-sensitive. Specifically, microstimulation delivered at over 400 sites produced echolocation pulses and communication calls, pinna flicking, tachycardia, and hyperventilation. Our data suggest that the BA and CA together constitute a higher, above the midbrain, control center for initiating vocalizations and modulating activity within multiple central pattern generators (CPGs) within the brainstem that control ongoing autonomic activity (Smith and Smith, 1989; Bellingham, 1998). Activation of these loci within the amygdala has the potential to orchestrate audiovocal output with behaviorally relevant physiological changes.

## Materials and methods

### Animal acquisition and maintenance

Wild mustached bats caught in Trinidad were housed in a room (3.5 × 1.5 × 2.5m^3^) with diurnal lighting conditions (light on from 09:00 to 14:00 h) in the animal facility at Georgetown University. The room walls were waterproofed and roughened and equipped with two dome-shaped structures (0.5 × 0.7 m) recessed into the ceiling with their inner surface clay-coated to permit roosting. During the light period, the bats congregated in the simulated “caves.” Environmental temperature was maintained at ~27°C and relative humidity at near 60%. Two feeding and watering stations were positioned on the walls. The bats were maintained on a diet of mealworms (*Tenebrio molitor*), enriched with vitamin and mineral supplements. The food and water dishes were changed daily. All husbandry and experimental procedures were approved by the Georgetown University Institutional Animal Care and Use Committee.

### Surgery and experimental set up

Six adult mustached bats (4 males and 2 females) ranging in weight 16–19 g were anesthetized with a subdural injection (0.18 ml) of 3.0% Domitor (medetomidine hydrochloride) to each animal 30 min before the surgeries. The skull was exposed while the bat was anesthetized with a mixture of oxygen and isoflurane gas (2–3%, about 700 cc/min O_2_). Bats were allowed to recover for 5–7 days after surgery before neural recording and behavioral testing were performed. Animals were placed in a soft Styrofoam holder to prevent escape and constrain gross body movements. The head was immobilized within a stereotaxic apparatus via a head post mounted on the bregma with light–cured dental adhesive (Kerr, Germany) onto. The body holder was suspended permitting neck and body movements relative to the head. All experiments were performed in a sound and echo-attenuating chamber with dimmed lights (darkness <10 lx). Small holes (80–100 μ in diameter) were drilled into the skull extending laterally 2600–3900 μm (mediolateral) from the midline bony ridge and 3200–4900 μm caudally from the frontal sinus at the intersection of olfactory bulbs and frontal cortex with the eyes and nostrils aligned in the horizontal plane. Adjacent holes were kept at least 100 μm apart. The head post and stereotaxic alignment was maintained across animals for all electrical stimulation and pharmacological procedures. During the experiment, a low-light sensitive video camera (BP334, Panasonic, Inc.) was used to monitor the animal and to observe behavioral responses, such as ear movements.

### Electrical stimulation and recording

Nearly all recordings were obtained during late morning to early evening hours with the bats in a resting state. Twisted nichrome wires with a tip diameter and tip separation of ~10 μm (1.0–2.5 MΩ) were used for electrical stimulation. The wire bundle was stiffened by a thin coating of Epoxy and inserted vertically into the dorsal surface of the brain with a Burleigh micropositioner. The tip of the microelectrode was carefully checked after each penetration for any unanticipated bending. The central and basal nuclei of the amygdala were targeted after careful localization using the methods described in Naumann and Kanwal (2011). A stimulation and recording session lasted from 3 to 6 hours depending on the ease of finding responsive sites. Spontaneous neural activity was monitored audiovisually as the electrode gradually descended into the brain. During neural recordings, unanaesthetized bats were placed in a Styrofoam body mold to prevent excessive bodily movement. The head-post was fixed in the same position and orientation across recording sessions relative to the axes of three perpendicular micromanipulators (Mitutoyo Digimatic 164 series). Stimulation site coordinates were recorded relative to a common origin on the stereotaxic apparatus across sessions, and reconstructed from marked lesions recovered from histologically processed brain sections.

Stimulation was accomplished with an isolated constant current generator (A365, WPI). Stimuli consisted of 0.5 ms monophasic pulses presented in trains of 100 ms duration at 100 Hz. Pulse train repetition rate was typically 5 Hz (sometimes 1 or 2 Hz). The typical stimulation period was 25 ms in each trial (duration of 1000 ms). The inter-trial interval was 60 s with a 0–30 s jitter; new trials were postponed if the bat showed any gross bodily movements. Stimuli were presented every 100–200 μm along a penetration. Current levels were usually between 5 and 40 μA (with an occasional maximum of up to 60 μA). The criteria for determining stimulus current threshold and detectable behavioral response were similar to the micro-stimulation techniques used in previous studies (Gooler and O'Neill, 1987; Schuller and Radtke-Schuller, 1990; Valentine et al., 2002).

Local field potential (LFP) activity was acquired from head-restrained animals before and after applying electrical and chemical stimulation. Signals were amplified and filtered between 1 and 300 Hz (RA16 medusa base station, Tucker-Davis Technologies, Florida) and recorded digitally at a sampling rate of 5 kHz using Spike2 software (Cambridge Electronic Design, CED). The electrode was slowly advanced into the brain using predetermined co-ordinates for BA and CA and relative recording positions were noted. Acoustic stimuli included 14 types of social calls (duration ranging from ~5 to 150 ms) played at a rate of 2/s. Electrical activity was recorded continuously for 500 ms. These recordings were also used to confirm a lack of physiological degradation of activity at the recording sites given the small amounts of electrical and pharmacological manipulation at the recording location.

### Pharmacological injection

Single–barrel glass micropipettes with tips broken to inner–tip diameters of <10 μm were used for iontophoretic injection of drugs. Micropipettes were filled with either saline (0.9% NaCl solution at physiological pH) or 0.1% KA dissolved in pH 7.3 PBS (Pillat and Schuller, 1998). The pipette was located stereotaxically in the BA and CA region prior to drug injections and remained in the place during iontophoresis and the recovery (Depaulis et al., 1989; Smotherman et al., 2006). At each stimulation site, saline was applied before injection of KA. A constant negative current between 20 and 40 μA was applied for 30–60 s prior to KA injection. No observable response was elicited during iontophoresis of saline using the same current as for KA. In addition, pressure injection of saline and KA (about 50 nl each) was also performed at a few sites to confirm responses obtained by iontophoresis.

### Measurement of EKG, and lung pressure

The electrocardiogram (EKG) was recorded differentially from two silver wire leads firmly pressed against the dorsal surface of each forearm. Bandpassed (3–30 Hz) EKG was amplified 10,000 times (Grass, model P55) and recorded at a sampling rate of 5 kHz with a Power 1401 and Spike2 software. Instantaneous HR preceding and subsequent to each stimulus marker was calculated using a custom–written script. Relative increase in HR was calculated by subtracting the pre- from the post-stimulation HR (6 s time window). More details about EKG data processing can be found in Ma et al. (2010). EKG was recorded from 3 bats roosting freely in a cage as well as when resting in the holder. No significant difference was observed in HR was recorded under these two conditions (9.10 ± 1.43 Hz, *n* = 3 bats) vs. those in a holder (9.25 ± 2.40 Hz, *n* = 3 bats) (Mann-Whitney U test, *P* > 0.05).

**Figure 1:**
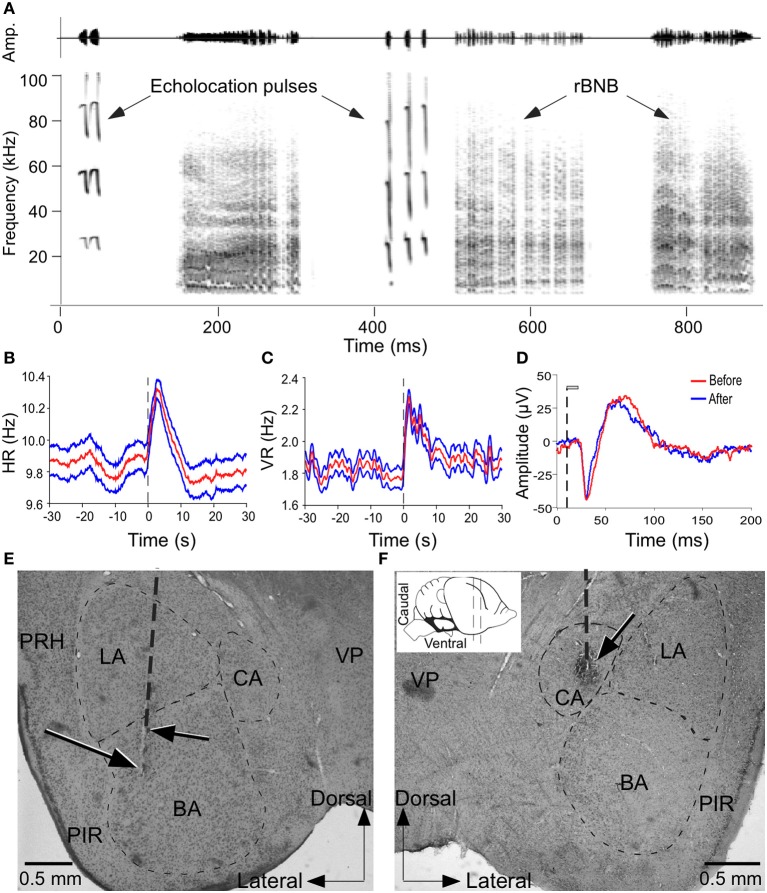
**Microstimulation of the amygdala elicited changes in autonomic and vocal activity. (A)** Amplitude envelopes (above) and spectrograms (below) of a sequence of echolocation and social vocalizations after electrical stimulation of the amygdala. **(B,C)** Plots to show increase in HR **(B)** and VR **(C)** before, during and after stimulation of a site in the amygdala. Both HR and VR continue to increase after stimulation and decline gradually returning to normal in 10–12 s. Vertical dashed line indicates stimulus onset at time 0. Red trace in middle (mean) is flanked by values for ± 1 standard deviation of the mean. **(D)** LFP traces to show the integrity of neurons at a stimulation site based on the similarity in the shape and peaks in the waveform before and after stimulation. LFP is average of 100 presentations of the same call before and after microstimulation. The vertical dashed line indicates the onset of acoustic stimulus and gray bar indicates call duration (20 ms). **(E,F)** Coronal brain sections with vertical dashed line indicating an electrode penetration and arrows pointing to lesions at stimulation sites within the BS and CA that triggered vocalizations and PM as well as increase in HR and VR. Inset shows a schematic side view of the mustached bat brain to show the plane of the section. VP, ventral posterior thalamic nucleus; PRH, perirhinal cortex; PIR, piriform cortex; CA, central amygdala; BA, basal amygdala; LA, lateral amygdala; ZI, zona incerta.

Changes in breathing were measured using a non-invasive method employing a custom-fabricated “pillow” (a 1.5 × 2.5 cm plastic bag filled with soft foam) of awake, unanesthetized bats. The pillow was placed in-between the chest cage of the bat (Smotherman et al., 2006) and the floor of the Styrofoam holder. Air pressure was transferred from the pillow to low-pressure sensor (model 1 Inch–D–MV, All sensors, San Jose, CA). Instantaneous breathing movements were amplified 950 times (Brownlee precision, model 400), digitized and recorded at a sampling rate of 5 kHz with a Power1401 and Spike2 software (CED). This provided analog recordings approximating the gross breathing pattern of the animal (Figure 1). Peaks in the breathing or “ventilation” pattern were used to compute VR prior to, during and after stimulation of the amygdala in awake bats. The procedures for this computation were the same as described earlier for EKG recordings (Ma et al., 2010). The resting VR was also monitored by a low-light sensitive video camera when a single bat was roosting quietly in the cage (30 × 20 × 25 cm). Previous studies used a whole-body plethysmograph to measure the resting VR, but for bats this measurement can be unreliable if a bats is not in its natural roosting position. To account for inherent changes in VR due to body position, we also recorded VR with the pillow placed gently under the chest when the bats were naturally roosting against the cage wall. No difference was found in the VR under these two conditions (2.15 ± 0.55 Hz, 3 bats) vs. roosting in a cage (2.12 ± 0.40 Hz, *n* = 3 bats; non-parametric Mann-Whitney U test, *P* > 0.05).

### Vocalization recording and analysis

Echolocation pulses and social calls were detected by an ultrasonic microphone and sampled at 250 kHz (9569, ACO Pacific, Inc). The microphone was placed directly in front of (distance of ~10 cm) and in line with the nostrils and eyes of the immobilized bat. Sound analysis was based upon a 512 pt FFT (Hamming window). Sonograms were calculated using Avisoft Pro (Version 4.51) at a frequency resolution of 488 Hz and a temporal resolution of 0.512 ms. Spectrotemporal characteristics of each sound were quantified using the frequency corresponding to the peak in the power spectrum, interpulse interval (IPI; from the start of one call to the start of next call) and call duration. Species-specific social calls were classified according to their spectral characteristics as described earlier using quantitative criteria (Kanwal et al., 1994). Echolocation pulses were recorded before performing surgery and prior to experimentation as control data for tracking any day-to-day variation in the average frequency of the predominant, second harmonic in echolocation pulses emitted at rest.

### Tracking and verification of stimulation loci

Stereotaxic coordinates were used to record the location of the electrode tip during the experiment. The BA and CA were targeted using an unpublished atlas of the mustached bat brain (provided by Dr. William O'Neill) and measurements from Nissl-stained coronal sections available from a previous study (Prasada Rao and Kanwal, 2004). Our stereotaxic method and apparatus generally follow those outlined by Schuller et al. (1986). Advancement of the electrode was also guided by monitoring the level of background neural activity. At some locations, LFPs were recorded (see above) to confirm auditory inputs to the region being stimulated and these were used as primary stimulation sites together with neighboring locations. The location of the electrode tip for recording and stimulation was marked with an electrolytic lesion (3 μA in 15 s with DC current) after the final stimulation in each bat and electrode penetrations were verified using histology. Cryostat sections (30 μm thickness) were subjected to standard procedures and histochemical staining was used to confirm the location of stimulated sites within the BA and CA. Histological verification of the recording site was possible only at the end of all experimentation on an animal, but coordinates of all recording sites were adjusted for any deviations from the presumed location of the tip of the recording electrode in all three planes.

### Data analysis

Statistical analysis was conducted using commercial statistical software SPSS 17.0 (SYSTAT, Inc.). A two-tailed test (at the 0.05 level unless stated otherwise) was used to determine significance. Parametric tests were applied to data that were normally distributed (one-sample Kolmogorov-Smirnov test, *P* > 0.05); otherwise non-parametric tests were used. Pre- and post-stimulus HR and VR values were compared using the paired-samples *t*-test. Wilcoxon test was used to check the duration, IPI, and frequency of the echolocation pulses emitted prior to and post-stimulation. Mann-Whitney test was used to compare the difference in HR and VR due to electrical and chemical stimulation with control data. One-Way ANOVA was used to test the occurrence of increase in HR and VR, and PM in any of the three dimensions (dorsoventral, rostrocaudal, and mediolateral) in BA and CA. Multivariate analysis of variance (MANOVA) was used to test for similarity of the acoustic pattern (multiple acoustic parameters) of the stimulation-elicited call with the normally produced versions. Means are given with their standard deviation (unless otherwise stated). The topographical organization of evoked response was assessed in individual bats as well as across animals (Valentine et al., 2002).

## Results

### General properties of evoked responses in the BA and CA

Electrical stimulation experiments were performed at 461 sites within the central and basal amygdala in 6 adult mustached bats. All of the data presented were obtained at stimulus levels close to threshold at each site except when testing the effect of increasing current levels on motor output. For our purposes, the basal amygdala includes parvo and magnocellular divisions of the basal nucleus and the accessory basal nucleus, and the central amygdala includes the central nucleus and its marginal zone as its boundaries are not clearly demarcated by fiber tracts. Vocalizations were elicited at 48% (224/461) of the sites tested within these regions of the amygdala. An example in which electrical stimulation triggered a vocal sequence containing both echolocation and social calls is shown in Figure 1A. Stimulation enhanced HR and VR at 85% (392/461) and 86% (395/461), respectively of the sites tested. Response dynamics for HR and VR are illustrated in Figures 1B,C, respectively. PM was elicited by stimulation at 79% (364/461) of the sites.

Data from one bat showed that lowering stimulation frequency (80, 60, 40, and 20 Hz) for suprathreshold current (~5 μA above threshold) intensity only slightly increased HR (0.20 ± 0.93%, *n* = 80 trials) and VR (0.62 ± 5.86%, *n* = 80 trials). In the 80 trials at 6 stimulation sites, HR and VR could be modified in 40 and 44% of the trials, respectively. These rates were significantly lower than those elicited at 100 Hz at the same sites (76 and 75% for HR and VR, respectively; *n* = 55 trials) (Chi-Square Test, *P* < 0.05). In addition, the occurrence of PMs at stimulation frequency (≤100 Hz) was reduced from 25 to 10% (8/80) for the same current intensity (Chi-Square Test, *P* < 0.05). Within these sites, three trials (5.5%, 3/50) elicited vocalization (echolocation pulses) with stimulation at 100 Hz, but no vocalization was produced when the frequency was ≤80 Hz. All data presented below were obtained at a fixed stimulation frequency of 100 Hz.

Figure 1D is a tracing of two LFPs superimposed to show that call responses of the neurons was not affected after multiple trials of electrical and chemical stimulation. Figures 1E,F show recording locations in the BA and CA, respectively that were marked by an electrical lesion. Microstimulation (20 μA current pulses) at these sites triggered vocalizations and PM, as well as increases in HR and VR.

### Changes in HR and VR

**Figure 2:**
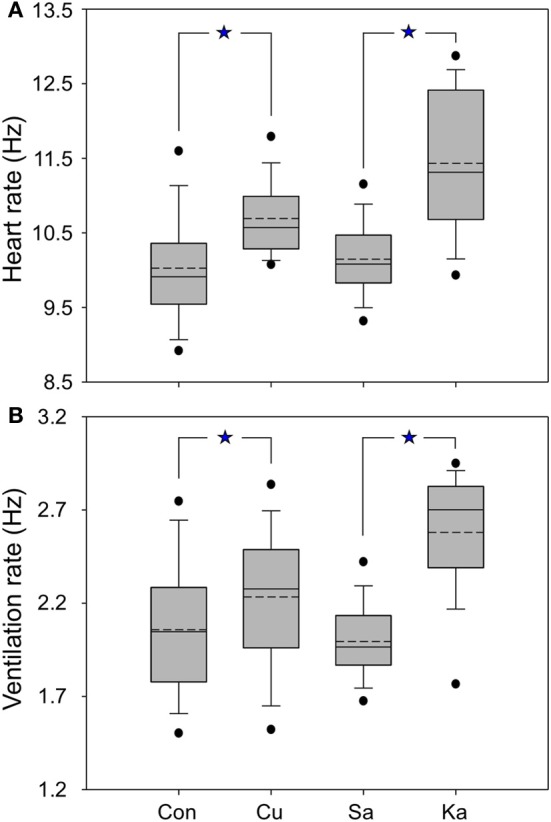
**Box plots showing the averaged heart rate **(A)** and respiration **(B)** of mustached bats for control and experimental conditions.** Control (Con) refers to the data collected before performing current (Cu) and KA stimulation; saline (Sa) means the data were collected after saline injection at the same site as electrical and KA stimulation. Dashed lines are means, solid lines are medians; boxes surround 1 *SD*, whiskers are 10th and 90th percentiles, and black dots are 5th and 95th percentiles. All data are normalized relative to the pre-stimulation HR and VR (see Materials and Methods). ^*^p < 0.05.

Both electrical and chemical stimulation resulted in changes in HR and VR. Increase in HR was observed for 85% of the sites for electrical stimulation and 76% (22 of 29) of sites for KA stimulation. Increase in VR was found at 86% electrically stimulated sites and 85% chemically stimulated sites (23 of 29 sites). Electrical current and KA produced significant increases in HR compared to control and saline injection (Mann-Whitney test, both *P* < 0.05) (Figures 2A,B). In addition, HR and VR increased rapidly from the baseline after the onset of current delivery, but with a latency of 20–30 s after the onset of KA delivery. The magnitude of changes was significantly higher for KA than for electrical stimulation.

### Production of echolocation pulses

Normal echolocation pulses of mustached bats start and end with transient frequency-modulated (FM) components and they usually include a relatively long constant-frequency (CF) element when vocalizing to detect a target or during roosting. A sequence of pulses in doublets and triplets are also sometimes emitted during roosting and when the animal is disturbed from rest. In our experiments, electrical microstimulation elicited either or both echolocation and social vocalizations at 49% of all sites within the BA and CA. Response latencies were distributed over a wide range and averaged 398.61 ms (± 215.17 ms), which included time for cycles of muscle contraction and relaxation, needed to generate complex sounds, as well as acoustic and electronic recording delays. Latency of evoked echolocation pulses (310 ± 90 ms (mean ± SD) was significantly longer than that for evoked social calls (mainly rBNB) 240 ± 60 ms (*P* = 0.032; independent samples, two-tailed *t*-test). The latency rarely changed with stimulus intensity but varied across stimulation sites, though not in any discernably systematic manner. Given the large variety of sounds generated, we did not analyze the latency data in detail since interpretation of these data was not very meaningful.

We pooled together all echolocation pulses from different bats from 122 stimulated sites. Each stimulated site elicited a different number of echolocation pulses ranging from a few to over a thousand. Given this wide range, we did not track the average number of pulses evoked at every individual site. The sample size in the results includes the total number of echolocation pulses analyzed from all of the stimulated sites. Among different stimulated sites, we did find obvious changes but no specific trend in the acoustic parameters of echolocation pulses. For this reason, we pooled all data together for quantification of results. Within 27% (122/461) of the sites, electrical stimulation yielded echolocation pulses and at another 6% (28/461) it elicited social calls. At only 16% (74/461) of all sites both echolocation pulses and social call sequences were produced in response to focal stimulation. On average, electrical stimulation elicited either 10 echolocation pulses or 4 social calls (range of 1–93 echolocation pulse and 1–16 social calls). In 2 bats, both KA and electrical stimulation were effective in eliciting vocalizations at 83% (*n* = 29) of the sites. All sites that yielded vocalization by KA injection failed to do so after a saline injection.

**Figure 3:**
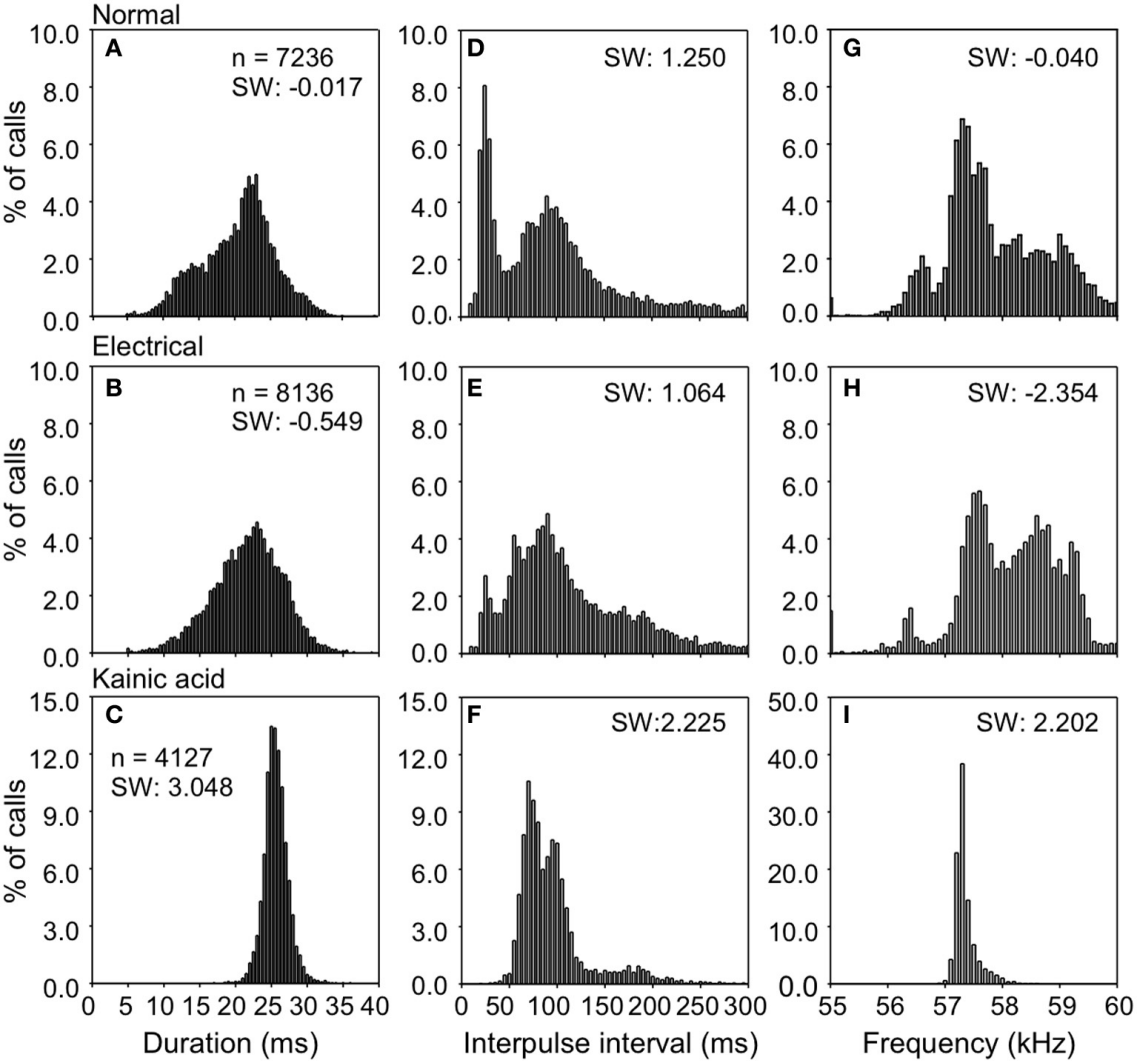
**Density plots showing the distributions for duration, interpulse intervals, and frequency of echolocation pulses elicited after electrical and KA stimulation.** Data were obtained from one bat; *n* is the number of analyzed pulses. **(A,D,G)** is for control; **(B,E,H)** during electrical stimulation; **(C,F,I)** during KA injection.

Figure 3 shows the distribution of duration, interpulse interval (IPI), and dominant frequency in natural and stimulation-elicited or evoked vocalizations sampled from all 6 bats. The mean duration of evoked echolocation pulses emitted at rest was 20.05 ± 5.48 ms. Naturally emitted echolocation vocalizations typically consist of solitary pulses, although doublets and triplets are also sometimes observed. The distribution of evoked pulse duration for doublets exhibited a shallow kurtosis for short pulses and a steep kurtosis for the relatively long pulses. Modes were observed at ~14.00 and 23.00 ms for short and long pulses, respectively (Figure 3A). Pulse duration for electrical stimulation-elicited vocalizations was slightly but not significantly longer (21.21 ± 5.26 ms, *n* = 8136), compared to natural vocalizations. Electrical stimulation-elicited echolocation pulse sequences that were mostly solitary exhibited a normal distribution for the duration parameter (Figure 3B). After KA injection, the bats usually started to emit echolocation pulses in 30–120 s and continuously produced sounds for 5–20 min. Evoked solitary pulses, when produced, were of relatively long duration (25.41 ± 1.89 ms *n* = 4127) with a narrow unimodal distribution for duration (Figure 3C). These data are summarized in Table 1. We did not find a significant difference in the duration of evoked echolocation pulses compared to the naturally emitted echolocation pulses. Chemical stimuli elicited pulses that overall were longer than those elicited by electrical stimuli. These data are for echolocation pulses only and do not pertain to social calls, which can be highly variable.

**Table 1:** **Parameters of echolocation pulses emitted under natural conditions and during stimulation at different sites**.

**Parameters**	**Condition**	**Mean ± *SD***	**Range**
Duration (ms)	Natural	20.05 ± 5.48	3.98–31.99
	Electrical	21.21 ± 5.26	3.66–28.27
	Chemical	25.41 ± 1.89	6.54–29.09
IPI (ms)	Natural	96.35 ± 71.39	11.58–300.13
	Electrical	111.56 ± 66.13	10.47–349.20
	Chemical	94.11 ± 39.47	17.31–341.39
Frequency (kHz)	Natural	57.92 ± 1.23	55.59–60.40
	Electrical	58.02 ± 1.10	53.80–59.59
	Chemical	57.31 ± 0.20	56.46–58.81

**Figure 4:**
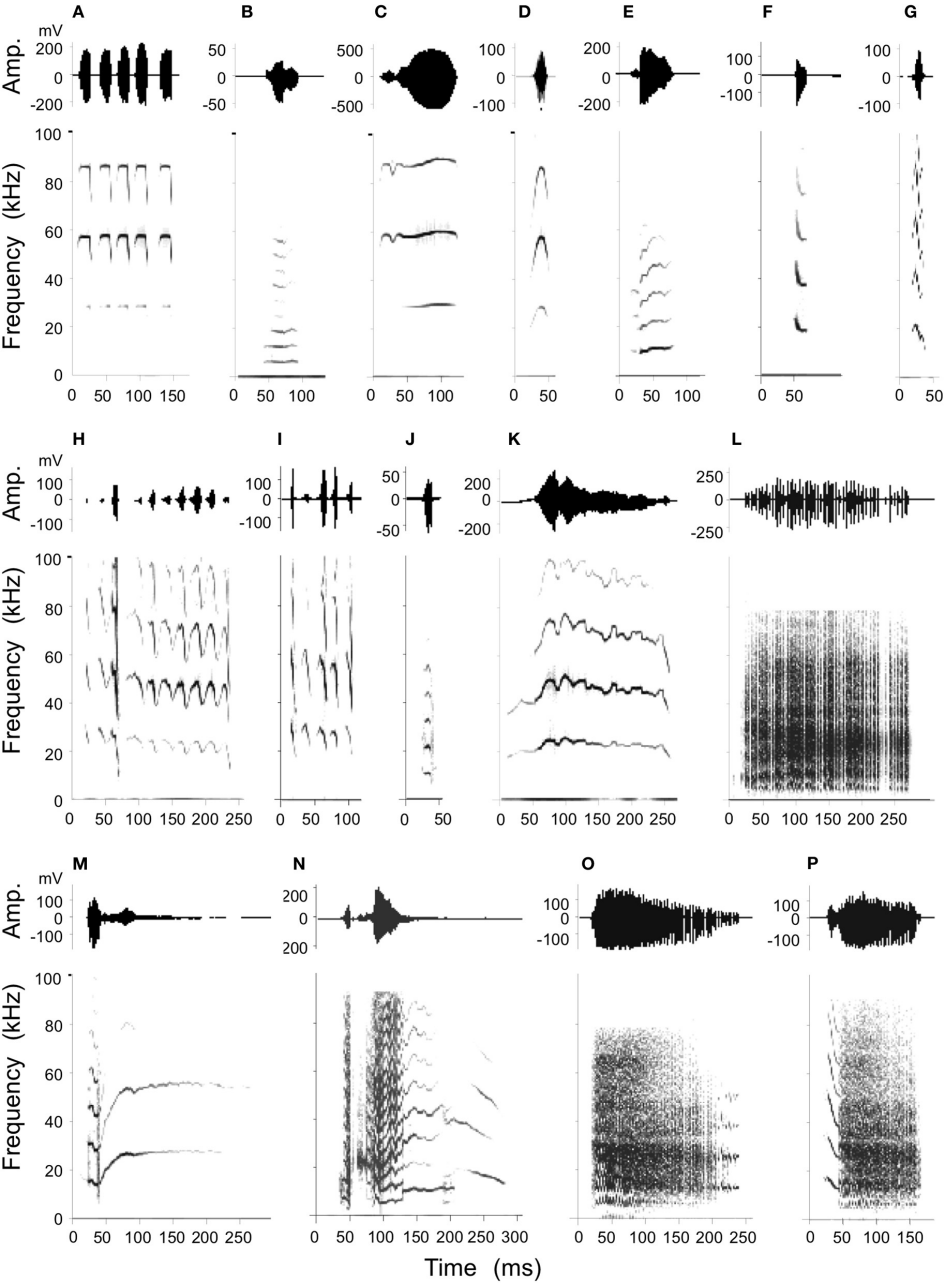
**Stimulation-elicited production of social calls including an example of echolocation pulses emitted at rest (A) for purposes of comparison.** Amplitude envelops (top) and spectrograms (below) are shown for each example. Call types produced consisted of simple syllables as well as composites as follows: **(B)**: short Quasi CF (QCFs), **(C)**: short true CF (TCFs), **(D)**: single arched FM (sAFM), **(E)**: bend upward FM (bUFM), **(F)**: bent downward FM (bDFM), **(G)**: single humped FM (sHFM), **(H)**: stretched rippled FM (sRFM), **(I)**: checked downward FM (cDFM), **(J)**: short wrinkled FM (WFMs), **(K)**: fixed sinusoidal FM (fSFM), **(L)**: rectangular broadband NB (rBNB), **(M)**: bUFM-long TCF, **(N)**: NB-DFM, **(O)**: NB-SFM, **(P)**: DFM-NB.

IPIs for echolocation pulses produced naturally in the resting state exhibited a bimodal distribution (Figure 4D). The IPIs exhibited a mean of 96.35 ± 71.39 ms. Short and long pulse doublets were separated by a relatively stereotypic short IPI, primary mode at ~25.00 ms (Figure 3D). The long IPIs between solitary pulses and doublets exhibited a second mode at ~90.00 ms (Figure 3D). The emission rate was lower for electrical stimulation-elicited pulses (IPI at 111.56 ± 66.13 ms) and the echolocation pulse sequence usually consisted of solitary vocalizations, doublets, or triplets. This pattern dramatically reduced the occurrence of IPIs at ~25 ms corresponding to the primary mode of the distribution for naturally emitted pulse sequences (Figure 3E). IPIs of echolocation pulses decreased even more after KA application (94.11 ± 39.47 ms). The mode was centered at ~70 ms and the distribution exhibited a smaller spread compared to that of the distribution for control and electrical stimulation elicited pulses (Figure 3H).

The dominant frequency corresponding to the second harmonic in the echolocation pulse was at 57.92 ± 1.23 kHz with a broad non-normal distribution centered at ~57.3 kHz (Figure 3G). After electrical stimulation, the distribution of dominant frequencies exhibited a peak at 58.02 ± 1.10 kHz centered at ~58.7 kHz (Figure 3H). After KA application, the mean echolocation pulse frequency was 57.31 ± 0.20 kHz and this parameter exhibited a very narrow, unimodal distribution (Figure 3I).

Compared to the control, electrical stimulation resulted in a distribution of duration, IPI and frequency that was slightly skewed to the left (skewness: −0.017 to −0.549, 1.250 to 1.064, −0.040 to −2.354). In contrast, KA application shifted the distributions of duration, IPI and frequency toward the right compared to the control (skewness: −0.017 to 3.048, 1.250 to 2.225, −0.040 to 2.202). However, the average duration, IPI or frequency did not differ significantly from control after electrical and KA stimulation (all *P* > 0.05, Wilcoxon test).

In general, the shape of the distribution of parameters values for electric stimulation mimicked those in the naturally elicited vocalizations, although both manipulations (electrical stimulation as well as application of KA) reduced the spread in the distributions of duration, IPI and the predominant frequency (second harmonic) in echolocation pluses (see Figure 3). Furthermore, the mean duration was longer and IPI's were shorter compared to echolocation pulses emitted naturally. The mean pulse frequency was slightly higher after electrical stimulation and lower after KA application, but the differences were not significant.

### Production of social calls

Previous studies show that mustached bats produce at least 19 types of simple syllable and 14 composites consisting of specific combinations of the simple syllables (Kanwal et al., 1994). A statistical analysis of these has resulted in a well-defined classification scheme based on the type of basic acoustic pattern (constant frequency, frequency modulation, or noiseburst) present within each call type and on the acoustic characteristics (e.g., fundamental frequency, predominant and number of harmonics, etc.) and/or the shapes of the frequency modulations within the spectrograms. Here, microstimulation at various sites within the BA and CA resulted in the production of 11 types of simple syllables and 4 types of composites (Figure 4). Visual examination of spectrograms revealed that each artificially elicited call type was remarkably similar to the naturally emitted calls (see Figure 1 of Clement and Kanwal, 2012) and therefore each call could be readily assigned to a specific simple syllabic or composite call type. Oscillograms of the amplitude envelopes also roughly matched their shapes within naturally emitted calls. The majority (85%) of all instances of call emission were of the rectangular broadband noise burst (rBNB) type. A multivariate analysis of spectrographic parameters (duration, upper and lower frequency bounds and center of predominant frequency band) in rBNB did not yield a significant acoustic difference (MANOVA; *F* = 0.156, *P* = 0.695) between normally produced rBNB and that elicited by electrical stimulation. Other call types were not as frequently emitted and therefore did not lend themselves to rigorous statistical analysis.

### Relationship between current intensity and behavioral response

**Figure 5:**
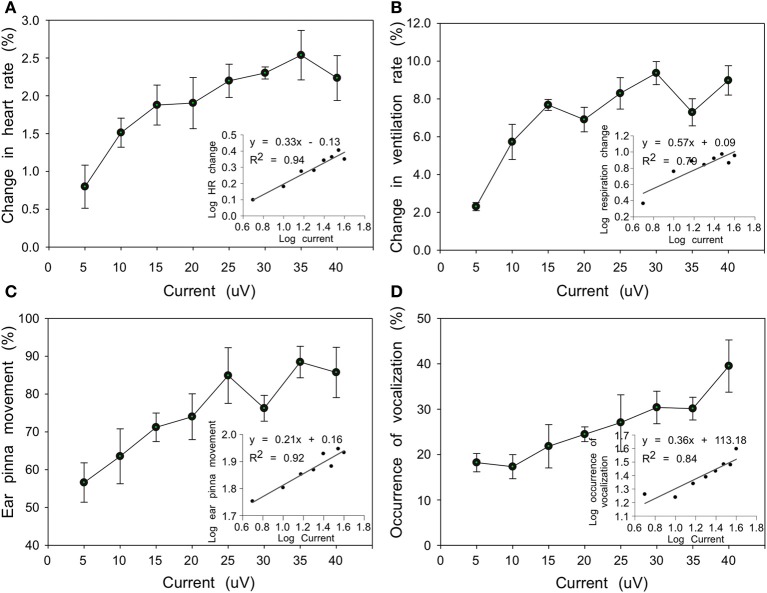
**Line plots showing relationship between current intensity and behavioral responses (A, HR; B, VR; C, PM; D, echolocation vocalizations) averaged from 4 bats.** Each dot refers to the averaged behavioral responses (%) relative to the total number of presentations in which a specific current intensity was applied. Insets in each plot show significant linear regression between log_10_-transformed current intensity and behavioral response.

At some stimulation sites, current intensities ranging from 5 to 40 μA showed a positive association with the behavioral response. HR gradually increased from 0.8 to 2.2% and VR increased from 2.3 to 9.0% corresponding to current increases from 5 to 40 μA. The VR appeared to be more sensitive to current stimulation (Wilcoxon test, *n* = 8, *P* < 0.05) and the average increase was bigger than that of HR with same current intensity (Figures 5A,B). At 79% (364/461) of the sites, evoked PM accompanied changes in HR and VR observed within the same penetration and for the same range of current intensities. For the same current range, however, vocalizations were induced at only 49% (224/461) of the sites (Figures 5C,D). Between PM and vocalization activity, the former was more sensitive to increases in current intensity (Wilcoxon test, *n* = 8 levels of intensity, *P* < 0.05). In addition, significantly linear positive associations were found between log_10_-transformed scales of current intensity and response frequency for behavioral responses (HR: *F* = 256.62, *df* = 6, *P* < 0.001; VR: *F* = 63.87, *df* = 6, *P* < 0.001; PM: *F* = 44.24, *df* = 6, *P* < 0.001; vocalization: *F* = 383.66, *df* = 6, *P* < 0.001) (insets in Figures 5A–D), establishing the physiological validity of observed responses.

### Spatial organization of vocal control

**Figure 6:**
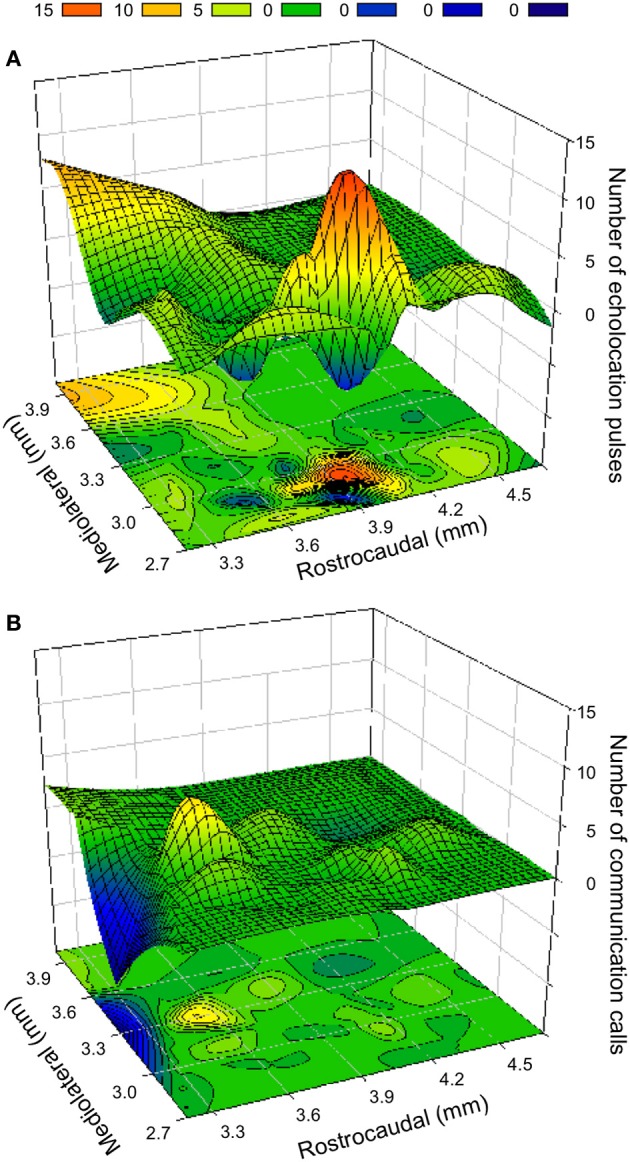
**Contour plots (below) and surface topographies (above) for frequency of vocalization elicited by electrical and kainic acid stimulation in the BA and CA region of the amygdala. (A)** Distribution of sites that elicited echolocation pulses. The bump in the foreground overlaps with the coordinates of the CA region, whereas the large peak is well within the boundaries of the BA. **(B)** Distribution of the sites that elicited social calls. The color scale for the frequency of call occurrence is indicated at the top. Negative points resulting from best fit function are equated to 0. The rostrocaudal boundaries of each plot are given relative to the position of the intersection between olfactory bulbs and frontal cortex. For obtaining stereotaxic coordinates, the eyes and nostrils are aligned in the horizontal plane. The depth is relative to the surface of the brain. The mediolateral distance is measured from the midline.

The topographical organization of evoked responses was assessed in individual bats as well as from data pooled across individuals to obtain a population estimate (Valentine et al., 2002). Tachycardia, hyperventilation and frequently PM were evoked in a broad area of BA and CA, but vocalization behavior was elicited in relatively specific areas. Echolocation pulses were easily evoked at the mediocaudal region of BA and CA (Figure 6A), whereas social calls were mostly evoked in a relatively rostrolateral area (Figure 6B). The distance between the two areas is about 300–500 μm and in the transition zone both types of sounds can be elicited. Contours were generated from computer-reconstructed localization of stimulation sites for each response type.

Along the vertical axis, echolocation pulses were elicited in a large part of the horizontal plane, but the occurrence (21.1%) in a range of about 1 mm (depths of 3.8–4.8 mm) was higher than at other depths (≤16.4%); however, the occurrence of social calls (on average 5.0–10.0%) did not show a clear preference along the dorsoventral axis. Mediolaterally, the production of echolocation pulses and social calls was higher over a range of 800 μm (from 2.7 to 3.5 mm) compared to other areas (19.6 vs. 15.2% and 11.2 vs. 3.6%, respectively). Rostrocaudally over a range of 1.2 mm (from 3.3 to 4.5 mm from anterior end of LA), echolocation pulses and calls were elicited with a frequency of 20.2 and 11.0%, respectively; in contrast to 14.8 and 6.2%, respectively, in other areas. Along particular dorsoventral and mediolateral planes, production of echolocation pulses was significantly higher than that of social calls (Independent-Samples *t*–test, both *P* < 0.05), but no significant difference was found along the rostrocaudal axis (Independent-Samples *t*-test, *P* > 0.05).

### Multimodal response localization

**Figure 7:**
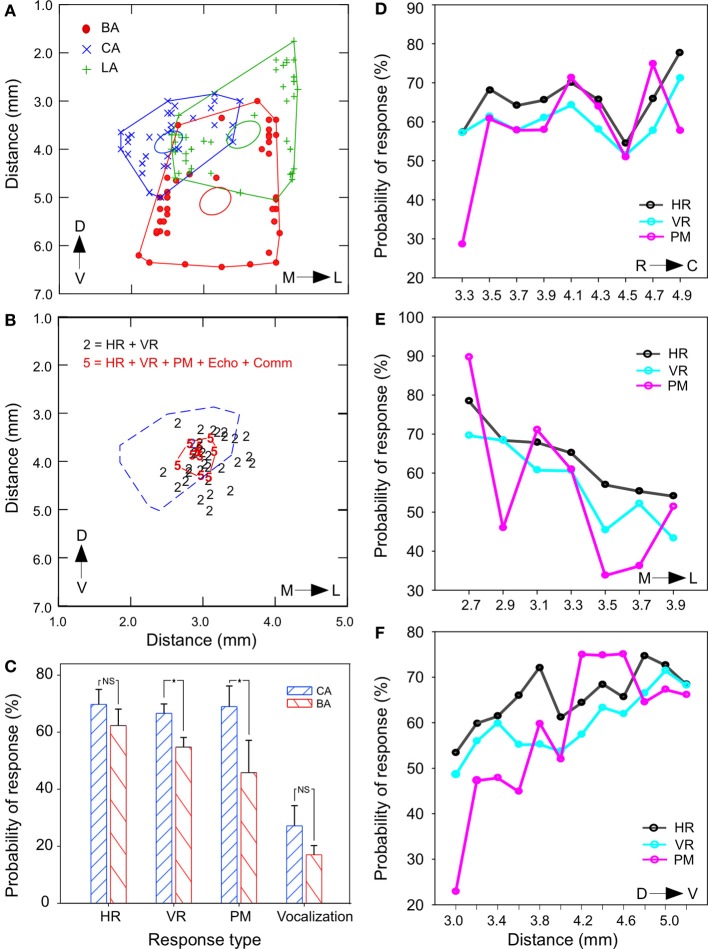
**Spatial distribution and overlap of multimodal response sites in the BA and CA. (A)** Scatter plots with lines connecting the outermost shell of BA, CA, and the LA on a rostrocaudally flattened map of the amygdala in one animal. Ellipsoids indicate the centroid (0.95 confidence level) of each scatterplot. Data points marked by symbols represent measurements at multiple rostrocaudal levels. The LA is relatively large at the anterior end and the BA covers a large portion of the amygdala at the posterior end. Mediolateral measurements are from midline and dorsoventral measurements are from the surface of the brain. **(B)** Location of stimulation sites that yielded all 5 modalities (labeled as “5”) of response output vs. those that yielded only 2 types of autonomic (HR and VR) output (labeled as “2”) for similar range of current intensity. Highly multimodal sites are tightly clustered and are contained almost entirely with the CA region (marked by dashed lines). **(C)** Bar graph showing that the probability of occurrence of stimulation induced changes in VR and pinna movements (PM) was significantly greater in the CA region. **(D–F)** Line plots showing a tendency or lack thereof for the probability of occurrence of response for each of the three non-vocal modalities (HR, VR, and PM) with coordinates localizing the stimulation site across all animals. Probability for occurrence of responses showed no clear trend along the rostrocaudal axis **(D)**, declined in the lateral vs. the medial regions **(E)** and increased along the dorsal to ventral direction in the BA **(F)**. ^*^p < 0.05; NS, not significant.

Detailed measurements from histological sections in one animal indicated the LA to be ~2.5 mm, BA to be ~2.0 mm, and CA ~1.55 mm. A rostrocaudally flattened map of the outermost coordinates of each nucleus obtained from several rostrocaudal levels (ranging over ~4 mm) are shown as a scatter plot in Figure 7A. The size of each nucleus varies along the rostrocaudal direction resulting in overlapping boundaries. The presence of multimodal responses for smallest current intensities (≤10 μA) at single stimulation sites was the overriding theme that emerged from simultaneous monitoring of multiple physiological measures in this study. At virtually all sites, different combinations of 3 response modalities were observed. Increasing current intensities sometimes resulted in the recruitment of additional modalities. For example, at relatively high currents (>20 μA) that exceeded threshold values for PM, microstimulation also led to emission of echolocation sounds (series of 10–20 pulses). Vocalization of social calls was almost always accompanied by increases in HR, VR, PM, and sometimes orofacial movements. Superimposition of response-combination types in a spatial frame-of-reference show that all 5 modalities were activated in a tightly clustered area in a central region that was contained largely within the CA (dashed red line in Figure 7B). Differences in changes are quantified in the bar graph in Figure 7C. Stimulation of the CA produced tachycardia, hyperventilation and PM more frequently than that in BA (Chi Square test, all *P* < 0.05). For 4 stimulation sites tested outside of the amygdala (caudate-putamen), stimulation at 100 Hz (10–40 μA) did not produce a significant increase in HR and VR (paired-sample *t*-test, *P* > 0.05, *n* = 50 trials). No vocalizations were produced at these locations outside the centrobasal amygdala.

Figures 7D–F summarize separately, along each of the three spatial dimensions, the changes in autonomic and somatic modalities in HR, VR, and PM composed from stimulation sites in all animals. The trend was not significantly different (One–Way ANOVA, all *P* > 0.05) between modalities when mapped for each dimension. The occurrence of tachycardia and hyperventilation gradually increased to ~20% from dorsal to ventral and from rostral to the caudal dimension, but decreased about 20% from medial to lateral dimension. For the dorsoventral axis, comparisons were made between the most dorsal and most ventral locations corresponding to the BA and CA, respectively.

## Discussion

This study represents a first step toward elucidating the representation of multimodal motor activity within the amygdala from the viewpoint of natural vocal and social behavior. Here we have shown that, unlike the LA, which is a major target of sensory inputs (Romanski et al., 1993; Naumann and Kanwal, 2011), the BA and CA constitute the neural substrate for life-preserving autonomic changes and accompanying vocal behaviors. Whereas early studies elucidated a role of the CA in modulation of autonomic activity (see below), the contribution of the BA was either considered to be minimal or not explored as well. Our results indicate that at stimulation levels close to threshold, multiple motor patterns occur together in the vast majority of locations tested within the BA and CA.

### Vocal control within the amygdala

The data from our study also suggest that natural vocalizations may be elicited by activity at any of multiple locations within the centrobasal amygdala. The production of echolocation pulses was more common than that of social calls. Vocalization parameters were more narrowly constrained after KA application than after electrical stimulation (see Figure 3). The distribution for each parameter was a subset of the distributions of naturally emitted pulses. Electric microstimulation depolarizes neurons depending on distance, degree of myelination, and proximity of electrode to spike initiation zone. Even for a small (~10 μA) current pulse, a greater diversity of neurons with dendrites and axons traversing a volume of ~100 μm^3^ (Stoney et al., 1968; Tehovnik, 1996), will be activated via electrical stimulation compared to KA application. The KA application is constrained to synaptic sites within the amygdala and the data suggest that activity within the amygdala likely controls a subset of the parameters of vocalizations. Accordingly, response parameters of vocalizations elicited by electrical stimulation can be expected to have a wider spread than those elicited after KA application.

The fact that the rBNB call type was the most frequently produced is consistent with the role of the amygdala in aggression (Clement and Kanwal, 2012). Accordingly, it is not surprising that this vocalization was almost always accompanied by increases in HR, VR, PM, and sometimes orofacial movements that constitute typical features of aggressive interactions. Not all locations elicited similar vocalizations, discounting the possibility that vocalizations are produced as a result of general arousal. The rostromedial region of the amygdala is primarily active in eliciting communication calls, particularly the aggressive rBNB call type, suggesting the possibility of an axis of arousal-to-aggressive activity within the centrobasal amygdala. This differential activation scheme is similar to that within the midbrain of *Phyllostomus* (Fenzl and Schuller, 2005; Liu et al., 2013) where electrical stimulation in the PLA elicited only echolocation pulses, whereas in the PAG either pulses or different call types could be evoked (Suga and Schlegel, 1973; Schuller, 1990).

### Vocal control pathways

The timing and nature of vocalizations can have immediate and important consequences for the emitter, such as during agonistic interactions with conspecifics and for echolocation in bats. Not surprisingly, the neural circuitry for vocal control in mammals is complex, requiring subsecond timing of vocal cords and respiratory muscles for continuous integration with multimodal, including auditory, feedback (Smotherman et al., 2006). Temporal precision in vocal output is accomplished via multiple upper and lower level loops and their interactions within specific brain regions (Jürgens, 2009).

**Figure 8:**
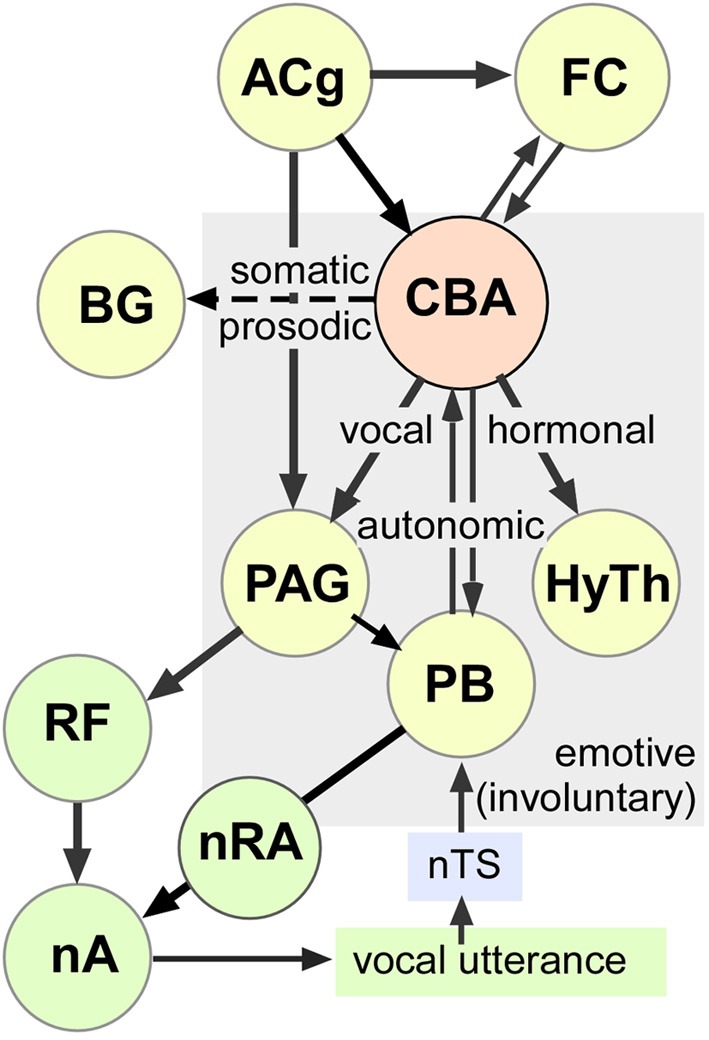
**Schematic-representation of an amygdala-centric control of emotive vocalizations within multimodal outputs.** This working model is based on studies of others as well as our observations of stimulation-induced vocal, autonomic, and somatic activity mediated by neuronal clusters in the central and basal amygdala in mustached bats. Long term hormonal changes (not measured here) are presumably mediated by the hypothalamus. The exact path by which each effect is mediated is less clear and could involve descending projections as well as higher-order loops through the cortex. Dashed arrow represents putative projection (evidence for specific projections and effects on prosody and pinna movements is lacking) to the basal ganglia, which also projects to the ACg via the thalamus. The schematic includes information from multiple sources (see text for details). AC, Auditory Cortex; ACg, Anterior Cingulate; BG, Basal Ganglia; FC, Frontal Cortex; CBA, Centrobasal Amygdala; HyTh, Hypothalamus; nA, nucleus Ambiguus; nRA, nucleus Retroambiguus; nTS, nucleus of the Solitary Tract; PAG, Periaquedectal Gray; PB, Parabrachium; RF, Reticular Formation.

Voluntary vocal activity can have a strong learning component involving activity in the basal ganglia, thalamus and motor cortex (Jarvis, 2007; Jürgens, 2009). Involuntary or innate vocalizations have predominantly emotive content. For their regulation, output pathways include the anterior cingulate (ACg), frontal cortex, and the amygdala (see Figure 8). The ACg projects to both the adjacent medial prefrontal cortex and to the motor cortex via the mediodorsal thalamic nucleus constituting a limbic loop for the control of vocalizations (Cardinal et al., 2002; Hage and Jürgens, 2006a,b; Jürgens and Hage, 2007). Similar to the central and basal amygdala, electrical microstimulation of the ACg elicits echolocation and social vocalizations in mustached bats (Gooler and O'Neill, 1987). Finally, projections between the amygdala and basal ganglia have the potential to modulate the prosodic structure of learned vocalizations according to emotive states (motivational drive, arousal, etc.) as well as elicit relevant somatomotor activity, which would enable simultaneous display of vocal and bodily gestures with high temporal precision (Jarvis, 2007; Feenders et al., 2008).

Cortical vocal control pathways are most extensively studied in primates, where they project directly to premotor neurons in the reticular formation, which contains a vocal pattern generator (Jürgens and Pratt, 1979a,b; Hage and Jürgens, 2006b). Lower vocal motor paths (from the PAG and the parabrachial region) are sensitive to auditory feedback and refine the spectral patterning of vocalizations embedded within the reticular formation prior to activation of phonatory motoneurons in the nucleus ambiguous (Jürgens and Ploog, 1981; Hage and Jürgens, 2006b; Jürgens and Hage, 2007). Integration with ventilatory muscle activity is presumably achieved via parabrachial nuclei, which receive inputs both from the PAG and from sensory feedback via the nucleus of the solitary tract. Parabrachial can either excite or inhibit inspiratory and expiratory motoneurons in the nucleus retroambiguus (Smotherman et al., 2003). The parabrachial nucleus is particularly important for the control of pitch in one's own voice, which is most highly developed for Doppler shift compensation in bats emitting CF containing pulses for echolocation (Smotherman et al., 2003, 2006).

Early studies in primates also suggested that pathways originating from the amygdala trigger vocalizations when stimulated electrically (Jürgens, 1982; Kirzinger and Jürgens, 1982). The spectrotemporal structure of these vocalizations matched those expressing either aggression or submission and showed some specificity to the stimulation sites. The importance of the amygdala in triggering vocalizations was discounted, however, because of an inability to trigger vocalizations by microdialysis of glutamate agonists into the amygdala (Jürgens, 2009). It was concluded that only combined stimulation at multiple sites leads to vocal action by the amygdala. Thus, in a recent model of vocal control, the PAG was implicated in the readiness to produce innate vocal patterns, such as non-verbal emotional utterances of humans and monkey calls, without the involvement of the amygdala (Jürgens, 1994, 2009). Our data prompt a reconsideration of this perspective.

Direct projections from the amygdala to the PAG, where vocal response latencies are a few tens of milliseconds (Fenzl and Schuller, 2002), cannot account for the relatively long vocal response latencies associated with single locus microstimulation in the amygdala. Rather, long vocal response latencies may result from the long lasting (~200 ms duration), excitatory and/or inhibitory call response patterns in the audiovocal basal amygdala (Naumann and Kanwal, 2011) that are not normally observed in purely auditory neurons elsewhere. Both, these response patterns and the long vocal response latencies may reflect recurrent activity intrinsic to the amygdala (Ehrlich et al., 2009) and/or persistent activity within either an audiomotor (thalamocortical) or a limbic loop involving the ACg where vocal response latencies are also a few hundred milliseconds (von Cramon and Jürgens, 1983). The wide spread of response latencies suggests that either the amygdala neurons or the circuits involved vary with the type of sound (echolocation or communication) or type of call elicited. Clearly, studies of simultaneously recorded spiking activity from multiple locations, preferably during naturally elicited vocal activity, are needed to further resolve these issues.

### Somatic and autonomic reflexes

Like other insectivorous bats that spend the day in a dark environment and hunt at night, in mustached bats too, visual acuity is poor but hearing is acute (Suthers and Wallis, 1970). Production of echolocation sounds together with PMs are integral components of echo localization (Schuller et al., 1997) whose initiation can be construed as an alerting response that allows a bat to become aware of its environment during feeding and social interactions (Clement and Kanwal, 2012). Ear movements in general are also associated with aggression and related social interactions as reported in other species (Hinde and Rowell, 1962; Buss and Estes, 1971).

The “central fear circuit,” within the amygdala (McDonald, 1998; Davis and Whalen, 2001) functions via connections of the LA with the basal and central amygdala (BA and CA) and its connections, in turn, with the lateral hypothalamus and target areas within the brainstem that directly mediate fear and anxiety (Rizvi et al., 1991; Pitkanen et al., 1997). Bradycardia and tachycardia following electrical stimulation of the amygdaloid region was first reported in squirrel monkeys (Reis and Oliphant, 1964). In cats, recordings of integrated activity in the phrenic nerve indicated that localized repetitive stimulation of the amygdala increased patterning of activity that corresponded to increased as well as decreased ventilation (Bonvallet and Bobo, 1972). These changes were frequently associated with cardio-acceleration and deceleration, respectively, when stimulating separately the parvocellular part of the BA and the peri-amygdaloid cortex (Bonvallet and Bobo, 1972). Stimulation applied to the magnocellular part of the BA (and in the anterior amygdaloid area) also provoked an immediate respiratory deactivation and cardio-deceleration, followed by a delayed respiratory activation and cardio-acceleration associated with cortico-ocular responses (Kapp et al., 1982; Applegate et al., 1983). These deceleration-acceleration phases of the respiratory and cardiac responses were suggested as being respectively related to the “attentive” or alerting and to the “defensive” stages of the defense reaction and were thought to be mediated via activity of amygdalofugal fibers running through the medial amygdalo-hypothalamic component of the ventral amygdalofugal pathway. Modulation of each respiration cycle was not the focus of our study, but these reports are consistent with our observations on the existence of respiratory (ventilation) control within the BA and CA.

Projections from the CA to the cardioinhibitory neurons in the medulla are ipsilateral and projections of the left or right vagal efferents to the heart innervate different nodal points. Here stimulation of the CA from either the left or right hemisphere produced similar increases in heart period (Healy and Peck, 1997). In awake rabbits, a low level stimulation of the CA produced bradycardia accompanied by changes in respiration (Applegate et al., 1983). The most sensitive sites were located within the medial region of the CA. The bradycardia response was most commonly an increase in VR and a decrease in tidal volume, as well as pupillodilation. The baroreceptor reflex triggered by natural stimuli as well as by electrical stimulation of the CA neurons in lightly anesthetized cats indicated that the integration of cardiovascular and behavioral responses during arousal is transient (Schlor et al., 1984). This is expected since cardiovascular adjustments are under the control of CPGs within a hierarchy of closed loop reflex mechanisms designed to maintain homeostasis. The amygdala-initiated baroreceptor reflexes are mediated via the rostral components of the lateral subnucleus of the CA that project to the cardiovascular region of the bed nucleus of stria terminalis (BST) and mediate in part the depressor responses to stimulation of the rostral CA (Schlor et al., 1984). Depressor responses elicited from the caudal CA, however, are not mediated through BST, suggesting that at least two independent pathways originate in the CA for control of cardiovascular reflexes (Roder and Ciriello, 1993). Rhythmic discharge patterns corresponding to cardiac activity were also observed in neurons of the BA and CA complex in conscious, freely moving cats (Lambertz et al., 1995). Although HR changes observed in our study were relatively small (~2% above baseline), these changes are significant given the fact that they occurred while the animal was at rest. The full range of normally observed HR and VR values include activity during either whole body movements or flight.

Autonomic responses observed in our study may originate from stimulation of efferents to the BA and CA or direct activation of non-visceral (e.g., auditory) input. Our stimulation paradigm was not designed to be phase-locked to the respiratory cycle; rather to mimic alerting sensory inputs. We observed a sustained acceleration of the VR with stimulation (see Figure 1C) and when tested this was positively correlated with the strength of stimulation (see Figure 5B). Furthermore, neurons that discharge in the awake state are shown to be different from those that discharge during the quiet sleep state (Zhang et al., 1986) implicating a change in the physiological state to be correlated with activity-shifts within neurons in the amygdala. Vice-versa, burst-firing of neurons, as with either electrical or chemical stimulation, within the amygdala may induce a shift in the physiological state (Rosen and Davis, 1988) as has been shown for bursting activity of single neurons within the cortex (Li et al., 2009). Delivery of pulse trains in our experiments may trigger focal bursting of neuronal clusters that may underlie both autonomic and vocal response patterns.

### Behavioral significance of multimodal control: vigilance and action

Electrical stimulation of the amygdala is known to produce an enhanced acoustic startle response by activating a fairly homogeneous population of neurons (Rosen and Davis, 1988). PMs are naturally associated with alerting responses for sound localization and during normal echo perception in synchrony with emission of echolocation pulses (Mogdans et al., 1988). Thus, the CA contributes to the production of an integrated and species-appropriate emotional response. Frequently, this response involves freezing under threatening conditions (Ciocchi et al., 2010). The most common response that we observed was tachycardia, acceleration of the VR and increased somatomotor activity, especially increase in PMs. These activations are most likely mediated via normal activation patterns within CPG circuits in the midbrain tegmentum and brainstem reticular formation since the acoustic structure of all of the stimulation-elicited echolocation and social vocalizations was similar to the ones produced naturally. A portion of these circuits may also lie in the pontine brainstem (Hage et al., 2006). Transitions in HR and VR were also smooth and somatic activity (PMs) corresponded to normal reflexive activity observed during either echolocation or social interactions in bats (Clement and Kanwal, 2012). These responses appear to be adaptations for vigilance and action either leading to insect-capture or reaction to an aggressive conspecific. They match the type of responses seen in predators such as cats (Stock et al., 1978) and contrast with those observed in prey species, such as rabbits (Kapp et al., 1982). Freezing and accompanying bradycardia in a flying predator, such as bats, would be non-adaptive when flying as well as when roosting in close proximity to conspecifics.

In conclusion, our data show that the amygdala contains multiple localized clusters of neurons dispersed within the centrobasal amygdala that can simultaneously activate different combinations of vocal, somatic, and autonomic motor activity. Further studies are needed to test whether the co-activation of “fight or flight” responses can reside within single neurons or requires the coordinated activity of local, overlapping circuits since extracellular stimulation does not permit isolation of less than 20 or 30 cells within the volume of stimulated tissue (Tehovnik, 1996). Most importantly, our data show that single site activation within the centrobasal amygdala can initiate vocal and even multimodal changes in motor output.

### Conflict of interest statement

The authors declare that the research was conducted in the absence of any commercial or financial relationships that could be construed as a potential conflict of interest.
